# Supplementary material for: A Mixed-Method Evaluation of a Rural Elementary School Implementing the Coordinated Approach to Child Health (CATCH) Program
Source: Nutrients. 2023 Jun 13;15(12):2729. doi: 10.3390/nu15122729 (PMC10304257; doi:10.3390/nu15122729)
Supplement: Supplementary file 1 [file nutrients-15-02729-s001.zip › nutrients-2377405-supplementary.pdf]

Why Measuring the Obesity Status of Rural NC Elementary School Children Matters: The Experience of a NC Academic Health Department

Carmen Samuel-Hodge, PhD, MS, RD[1]
Tyler George, BSPH;[2] Lisa Macon Harrison, MPH;[3] Kathryn Bernstein, MPH, RDN;[2] Tyisha Terry, MSPH;[3] Lindsey Bickers Bock, MPH;[3] Shante Speed, BSPH;[3] Jennifer Grable, MAE;[4] Kathrine Noel, RN;[5] Trevor Hamlett, BS, BA;[2] Ziya Gizlice, PhD; [2]Aurore Victor, JD[2]

[1] UNC Gillings school of Global Public Health, Chapel Hill, NC; [2] UNC Center for Health Promotion & Disease Prevention, Chapel Hill, NC; [3] Granville-Vance District Health Department, Oxford, NC; [4]NC Cooperative Extension, Oxford, NC; [5]Oxford United Methodist Church, Oxford, NC

Introduction

Like most other states, North Carolina (NC) does not mandate childhood obesity surveillance. Consequently, available data at the county level for prekindergarten and elementary school children is limited by small sample sizes and inclusion of only children participating in federally funded health and nutrition programs. Although rural children generally have higher rates of overweight and obesity compared to their metropolitan counterparts, making the case for action in under-resourced rural communities is challenging without local data.

Methods

- A district health department serving two rural counties, with funding through a local wellness coalition and evaluation support from an academic partner, measured heights and weights of children in public pre-kindergarten and elementary schools.
- Health department staff, university students and community volunteers were trained to measure height using a stadiometer, and weight with an electronic scale.
- Two values for each measure were recorded and averaged. Body Mass Index (BMI) percentiles and overweight and obesity status were determined using standard methods.

Results

- **N=4214**: 52.8% Non-Hispanic Blacks | 24.8% Non-Hispanic Whites | 17.7% Hispanic | 4.7% Other race/ethnicity
- Overweight: **42.4%** | Obesity: **24.9%**
- Hispanic children had significantly higher rates of overweight and obesity (49% and 29.9% respectively), compared to non-Hispanic Blacks (42.9% and 26% respectively) and non-Hispanic Whites (36.4% and 18.2 respectively).
- Obesity rates were not significantly different by gender, but were significantly different by grade, with the highest rate of obesity (34.7%) among 5th graders.

Figure S1

In rural communities, **local data** collected by community members can spark **community action** to promote healthier weight in children and demonstrate the value of a **rural academic health department**.

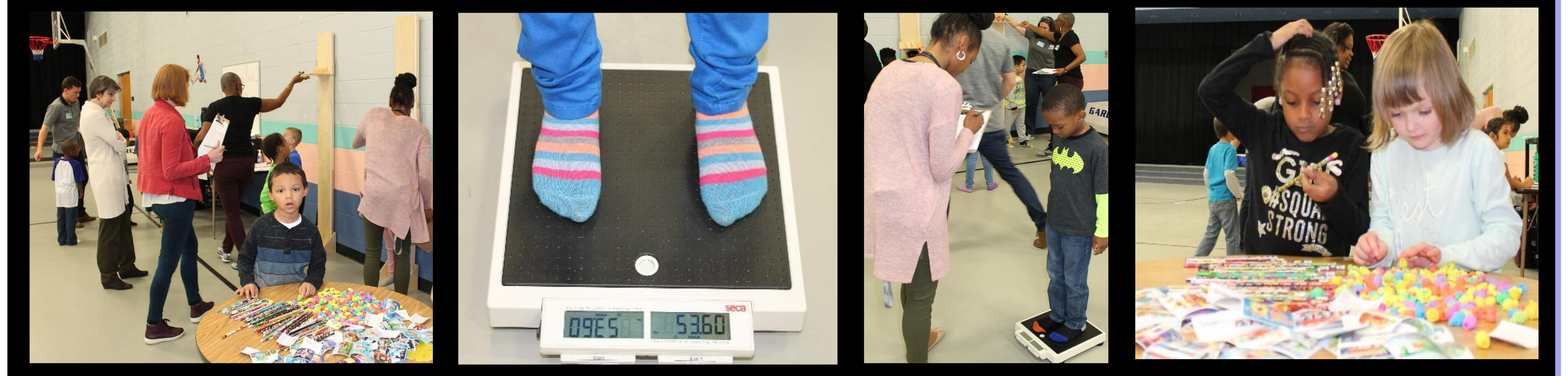

SCAN ME

Working on Wellness | Triangle North Healthcare Foundation | Granville Vance public health | UNC Gillings School of Global Public Health

Pilot Testing
- Measurement time & sequence of activities
- Staff needed

School Invitation
- Contact principals & share process for visit
- Provide parent letter & permission slip

School Follow-Up
- Send summary of results
- Provide school incentives

(N)	Overweight N (%)	Obesity N (%)
Total (4215)	1782 (**42.4%**)	1049 (**24.9%**)
Boys (2126)	889 (41.8%)	554 (26.1%)
Girls (2085)	895 (42.9%)	493 (23.6%)
NH-White (1047)	381 (36.4%)	191 (18.2%)
NH-Black (2224)	954 (42.9%)	579 (26.0%)
Hispanic (745)	362 (**48.6%**)	223 (**29.9%**)
Other (198)	87 (43.9%)	57 (28.8%)
**National Rates** (**6-11 y; 2015-16**)	**34.2%** **B** 37.7%, **G** 31%	**18.6%** **B** 20.7%,**G** 16.7%

Academic Health Department
- Public Health Education & Training
- Research
- Service

GRADE (N)	Obesity N (%)
Pre-K & K (993)	204 (20.5%)
First (675)	127 (18.8%)
Second (777)	186 (23.9%)
Third (758)	207 (27.3%)
Fourth (734)	230 (31.3%)
Fifth (277)	96 (**34.7%**)

Table S1: Domains, Themes, and Illustrative Quotes from Student Focus Groups and Interviews with Staff and Parents/Caregivers

Students (Focus Groups)

Domains and Themes	Illustrative Quotes
**A. CATCH Program Knowledge**<br>**A1. Most students positively recognize CATCH goals as related to healthy eating and exercise.**	"It's trying to make us exercise more." (FG2)<br>"Help your health." (FG2)<br>"That our principal asked us to eat healthy and get at least an hour a day outside and exercising." (FG3)<br>"It's like teaching us how to like exercise more, play more." (FG3)<br>"That it's nutritious stuff. Of food, it has something to do with food. " (FG4)<br>"It's about how you exercise and how you eat." (FG4)<br>"I don't know anything about it." (FG 1)<br>"I've never heard of the CATCH program" (FG1)
**B. Student Recommendations if they were the "Boss" of CATCH program**<br>**B1. Recommendations combining Healthy Eating with Physical Activity**	"So I would tell them to have at least one junk food a week but the rest of it healthier….have a big playground for them to run, jump on. And you shouldn't just sit down and talk at recess. Recess is a time to get exercise." (FG1)<br>"I think that teacher should allow every child to quickly walk, run or job the track at least once every day at recess and to not eat a lot of sweets." (FG2)
**B2. Students recommend increasing physical education/recess and games/competition.**	"More recess." (FG2)<br>"Might have an extra recess." (FG4)<br>"PE every day." (FG3)<br>"You could expand our PE time, physical education." (FG4)<br>"Work competition where they have to exercise a lot and whoever wins gets like field day." (FG2)<br>"If they get them to run 100 miles for $100." (FG2)<br>"Give ideas for games, like for recess so you're not just sitting down and talking the whole time." (FG1)<br>"I would help about a game that they might play and it would have exercise in it." (FG3)
**B3. Recommendations on Food and Meals**	
**B3.1 Students recommend**	"A little bit more baked foods and more vegetables and

**to include healthy foods, fruits, vegetables, and some of their favorite foods.**	fruits so you don't have so many fried foods with so much grease.” (FG1)<br>“I’d make they try to eat-I mean try a little healthy food every day.” (FG3)<br>“I would put healthy food in like the food for breakfast, healthy food in it and sneak it in there and put it in like – because then – I don't know. “ (FG4)<br>Nachos?” (FG3)<br>“Let’s see, there’s broccoli, and corn." (FG3)<br>“Apples and some oranges.” (FG3)<br>“Strawberries and tomatoes.” (FG4)<br>“Corn”, “avocado”, “bananas” (FG4)<br>“Apples”, cucumbers, “broccoli without the dang cheese”, “boiled taters”, “pickles” (FG4)
**B3.2 Students recognize and recommend schools should not include desserts, candy, and highly processed foods.**	“Candy”, “cake”, “salt”, “ice cream”, “cupcakes”, “soda”, “suckers”. (FG3)<br>“Probably not so many sugar snacks.” (FG1)<br>“Pancakes”, “pizza all day" (FG4)<br>“I wouldn't let them serve no cheesecake.” (FG4)<br>“I wouldn't let them do the funnel cakes”, “I would not let them all do the Cheez-its anymore”, “donuts”, “fried chicken". (FG4)
**B.4 Students mostly recommend discussing CATCH with Parents/Guardians and encouraging action.**	“I would tell them about the Go, Slow, Whoa food thing.” (FG2)<br>“I would want them to know like Go Slow Whoa thing here.” (FG3)<br>“It’s good for your health and weight.” (FG1)<br>“It’s not just good for you, it’s fun.” (FG1)<br>“Oh, I would want my parents to know that it’s actually helping everybody and if you want to be healthy you should maybe start it too.” (FG2)<br>“So I’d tell them to try to get up and go outside and run around….” (FG4)<br>“I would tell my grandpa for one: get his butt out of the recliner and hang out with us outside.” (FG4)<br>“Nothing.” (FG4)<br>“I would not let them know” (FG2)
**C. Go, Slow, Whoa (GSW)**<br>**C.1 Most Students express understanding of GSW**	“Go means maybe eat all the time; Slow means don’t eat as much as you should; and Whoa means just don’t eat it

	alot." (FG1)<br>"Whoa is like really bad for you. Slow is okay to have sometimes and Go is really good for you." (FG2)<br>"Kind of like they said: Go is something you should have every day; Slow is something you should have not a lot of but every once in a while, and Whoa is something that you wouldn't eat every day; you should eat it not very often. " (FG3)<br>"Nothing." (FG4)<br>"Go food is healthy food, Slow food is in the middle, and Whoa food is like candy …and stuff." (FG4)<br>"Whoa food means it's like healthy and then Slow food bad, and then – well yeah, and then -- " (FG4)
**C.2. Students observed GSW posters and information extensively throughout the school.**	"Cafeteria", "In the library.", "Hallway." (FG1)<br>"I seen it in my classroom." (FG1)<br>"We see it going down the hallway. There's like two on each hallway." (FG2)<br>"At the cafeteria and there's the My Plate thing." (FG2)<br>"I see it in the gym." (FG2)<br>"You can see it when you're walking down the halls by the bathrooms near the cafeteria or near PE. You see little charts that talk about Go Slow and Whoa foods." (FG3)<br>"On the walls, on the cafeteria." (FG4)
**D. Changes in Classroom**<br>**D.1 Energizers/Brain Breaks**<br>**D.1.1 Many students enjoyed brain breaks to increase PA, but liked some activities more than others.**	"Some of them I like but some of them I don't because some of them it's more–it's not as much movement as it is of some of it." (FG1)<br>"I like doing those because some people they like to dance and some of them don't." (FG1)<br>"When we do brain breaks we have this website called Granoodle [*sic*] and some of the stations are not so good and some of them are okay and some of them are great." (FG2)<br>"I like them, but the ones I don't like is the Kids Bop [*sic*] because the whole class will start singing it and it gets annoying." (FG2)<br>"It's really fun to watch everybody dance in our classroom." (FG3)<br>"We just take one little break, in the middle of our thing, and it helps me not think about reading and math that we have to do. It helps me give my brain a break, so I like it." (FG3)<br>"I think some of them are moving a lot, but some are too

	educational." (FG4)<br>"I think you should move more, and even if you're learning you should just move more." (FG4)<br>"Change everything because it's all wrong." (FG4).
**D.2 Lessons on GSW**<br>**D.2.1 While some students found lessons helpful, increasing active discussions may facilitate effectiveness for other students.**	"I like them because like it teaches us things that we can do to keep ourselves healthy and like if we – like if we – I don't know, it's hard to explain. But like I like it because it helps me learn." (FG2)<br>"No." (FG3)<br>"I don't like how like you just put up a poster and you don't hardly talk about it, all you do is look at it, you don't give no – you don't talk about it. You might say a few words on the intercom but nothing big. " (FG4)<br>"It tells kids that they should eat healthy stuff and not a lot of unhealthy crap but they can eat that unhealthy crap sometimes but not all the time." (FG4)
**E. Changes in eating because of GSW/other**<br>**E.1.1 Students made healthier choices based on GSW and were more willing to try new food.**	"It gave me examples, like if I'm trying to figure out if a cookie go into Whoa or Slow I could come to school and I could look on the chart and it gives examples and it could tell me which one it goes to." (FG1)<br>"...I didn't like spinach when I was little but then I now learned-when I learned about the Go Slow Whoa foods I liked it." (FG2)<br>"It encouraged me to try new things." (FG2)<br>"I like eat a little bit more of the Go foods*[inaudible]* eat." (FG3)<br>"Sometimes my mommy ____ chocolate to school for a little dessert after lunch. So she used to send me like two. But now she sometimes only sends me one." (FG3)<br>"It's made me think I should be eating more healthy and not eating like a bunch of cake all at once." (FG4)
**E1.2 Students positively associate Go foods with better physical performance.**	"But then when I started eating a lot of food like good food it was easier for me to play baseball…" (FG2)<br>"It actually helped with doing stuff when I was in sports. So when I play football I wasn't that fast of course, but then I started eating some healthier foods and then I actually got a lot faster." (FG2)
**E1.3 Some students made no changes to eating.**	"It didn't really change because I kept eating the same thing every day because it didn't really affect me." (FG2)<br>"It haven't" (FG2)

**F. Changes in Physical Education**<br>**F.1 Recess**<br>**F.1.1 Students expressed frustration at inactivity by some students during recess and due to shortened recess time.**	"I don't like the point that sometimes people sit down on the benches. I think that everybody should get up and play." (FG1)<br>"If you're sitting down on the benches I think you would rather go play and then when you get enough of your exercise and energy you should go sit down for a little while." (FG1)<br>"They stop letting us have extra, like and we supposed to have at least 40 minutes or 30 minutes of recess. But our class we only have at least 20 minutes to play." (FG4)<br>"Yeah, sometimes we don't even get more than like 10 minutes." (FG4)<br>"We never get to go outside." (FG4)
**F.1.2 Length of recess and level of activity may be influenced by teachers.**	"I don't really think it's a change of recess but I think it's probably a change of teachers because last year we had to walk laps almost every day at recess unless we went to go play kickball. But this year I really like our teachers, but we just don't walk a lap every day, we just go play. " (FG2)
**F.1.3 Some students observed same or increased physical activity at recess when compared to prior year.**	"Same." (FG3)<br>"Last year we really didn't walk a lot so this year we're walking like every morning if it's not raining. " (FG3)
**F.2 PE**<br>**F.2.1 Students positively responded to the variety of sports taught, but expressed frustration by decreased level of physical activity/playing in PE.**	"Last year we weren't learning how to do a lot of sports like baseball. This year we're learning volleyball, basketball, and I think we're going to learn more this year. " (FG3)<br>"I like PE now because like the other years they would say that you're not old enough to play that game and then you don't know how. And sometimes when you do, like when you do – like if you're in kindergarten you can't do a lot of stuff because you don't know how to do certain games or how to play it. " (FG1)<br>"I dislike it because it feels like they shortened it a little, and they don't do as many warmups, so then my legs started to feel stiffer when we was doing a running exercise-game thing." (FG1)<br>" Some of the students in our class when we do running relays they just walk instead of running." (FG1)

	“Basketball, football and baseball.” (FG4)<br>“We should play some games. This year we’re actually practicing a lot of sports; he’s teaching us how to do basketball and volleyball. It’s a lot of sports right now we’re learning. We’re not playing physical games.” (FG2)<br>“I see people all the time in gym; they always just sit there and stare at people, and they don’t even do stuff.” (FG2)
**F.2.2 Students response when asked if changes to PE from prior years**	“The same.” (FG2)<br>“It’s different.” (FG2)<br>“No.” (FG3)
**F.3 Fit Friday**<br>**F.3.1 Some students positively respond to Fit Friday health tips and physical activity.**	“I like Fit Friday because she gives us examples of things we should eat and how many minutes a day we should exercise and a bunch of stuff. “ (FG3)<br>“I like it because we get to exercise.” (FG2)<br>“Some people wear joggers, and it’s hot, and when they wear joggers it gets really–it’s hotter than it would if they wear something other.” (FG1)
**F.3.2 Fit Friday reception may be influenced by teacher participation and modeling.**	“...well last year my teacher was a man teacher and he would wear tennis shoes and he would actually exercise with us and play kickball with us every Friday. But for this year I just feel like my teachers wear tennis shoes. Sometimes they don’t wear tennis shoes and they forget and I don’t like we do exercise anyway.” (FG2)
**F.3.3 Some students did not recognize any changes/differences on Fit Friday.**	“Fit Fridays aren’t really Fit Fridays. They don’t really - it’s like the same.” (FG2)<br>“It's nothing that we do on Fit Friday.” (FG4)<br>“I never noticed Fit Friday.” (FG4)
**F.3.4 Some recommend more physical activity during Fit Fridays**	“For Fit Friday they could do something different like they could let us go out at recess for ten or five extra minutes.” (FG4)<br>“Thirty extra minutes of recess.” (FG4)

Parents/Caregivers

Domains and Themes	Illustrative Quotes
**A. Background Information**<br>**A.1 Parent/guardian's children attended Stovall for one up to five years.**	"Well, my son has been at this school for this academic year only." (090)<br>"This is his first year." (095)<br>"She was there for half of kindergarten and all of first grade and then all of fourth and fifth." (096)<br>"One year" (102)<br>I have one that's still there. He's been there for five years." (143)<br>Since kindergarten, and she's in the third grade, going into the fourth." (153)<br>"This child since she was in kindergarten. So that's what, two years?" (156)<br>"Three years." (158)
**A.2 Most of the parents and their children live outside of Stovall.**	"We do.." (090)<br>"No. We live in Oxford." (095).<br>"No. Actually, our mailing address is Oxford, but we are closer to the school than to actually Oxford City." (096)<br>"No. I live in – well, my address is technically in Oxford, but I'm much closer to Stovall than I am to Oxford." (102)<br>"We live in Bullock." (143)<br>"No. We live about six miles from there." (153)<br>"We live in Bullock." (156)<br>"No. We live just north of Stovall about ten minutes in a town called Bullock." (158)
**A.3 Parents value the small class sizes, health focus, as well as the community and student-centered atmosphere of Stovall.**	"I think he gained 18 pounds in the last year. Then he went to Stovall and lost it all back." (090)<br>"Well, the fact that the focus is on children and not testing." (090)<br>"It's more of a rural area school. The class sizes are smaller." (095)<br>"I like the fact that all the teachers know all the children and they care about the children whether they are there students or not." (096)<br>"I like that it's very close to my neighborhood. I like the teachers, and the principal, and the administration." (102)<br>"Just everything." (143)<br>"Like the teachers respond to the student if – they care about the students, not just getting the test scores for the school." (153)<br>"Well, everybody's a close family. Everybody, they're the same neighborhood, so we kind of pretty much know each other. I can talk to the teachers and pretty good communication." (156)<br>"Just small classroom sizes. I like that it's a community-based school. Everybody knows everybody type

	atmosphere. It's just a personal connection from the teachers to the kids.” (158)
**B. Perception of CATCH Program**<br>**B.1 While some parents expressed CATCH goals, others had not heard about CATCH.**	“Well, I know that a lot of it is about the healthy lifestyles….It’s not just go exercise today. It’s the continuous movement…They talk about healthy lunches.” (090)<br>“You know actually, all I really got about it was an email, to be honest. I honestly did not read through it.” (095)<br>“Tell me what it means because I'm not sure.” (096)<br>“I believe that the CATCH program is designed to encourage the kids who attend school – and probably the teachers, too – to teach them about the nutrition, to teach them about the importance of exercise, and how to eat and exercise in healthy ways…(102)<br>“I haven't really heard anything yet." (143)<br>**After facilitator explains CATCH:** “Yeah. I think I remember some of that." (143)<br>“I don't remember exactly what I had heard about it. I just know that when I heard about it, I was happy about it." (153)<br>“That's the exercise program, right?” (156)<br>“Yeah. They're encouraging the kids to do exercise, and tell about the different kinds of foods, and they're moving a certain length of time every day, stuff like that." (156)<br>“From my understanding of it, it's a healthy eating type program as far as what foods to eat, and how a child benefits from it, and what times of the day they should eat them, and how often they should eat, from my understanding of it so far.” (158)
**B.2 None of the parents/guardians played a role in bringing CATCH to Stovall-Shaw.**	“No. I'll be honest with you. I had a lot of apprehension. I thought it was just another gimmick, to be honest. I know it probably sounds terrible to say that.” (090)<br>“No, he [the child] just moved there for his fifth grade year.” (095)<br>“No." (096)<br>“No. I'm afraid I didn't. I found out about it when, after the kids were going to be attending, just because we were in the area, though I certainly would have considered the school, even if we hadn't been in the area." (102)<br>“No.” (143)<br>“No, ma'am. I've just been very happy about it.” (153)<br>“No.” (156)<br>“No. I did not.” (158)

**C. Changes in Physical Programs/Education**<br><br><br><br><br>**C.1 PE**<br>**C.1.1 Parents observed children enjoying more variety and activity in PE since CATCH.**<br><br><br><br><br><br><br><br><br><br><br><br><br>**C.2 Recess**<br>**C.2.1 Parents generally observed more equipment for and walking during recess since CATCH.**	**Both PE and Recess:** "He talks about….they're playing basketball. They're playing volleyball. They always were before, when they go outside for recess, they always have to walk at least two laps before they can go play….If you're not going to play, then you walk laps. You're going to be active." (090)<br><br>"Well, she just always – she's mentioned games and stuff that they play, and stuff they do. And it's always a deal of do I wear tennis shoes today or sandals? So we always keep up with that." (158)<br>"As far as PE, I've heard him talk about baseball and that was different." (095)<br>"Now he was talking about he is actually playing games and stuff like that. It's a difference." (095)<br>"I guess no. They do tell me about the activities and stations they do in PE and the different games that they play. I hear about the games they play fairly consistently. It doesn't seem that different from what I had when I was in PE." (102)<br>"She's done more like the jumping jacks, the running. She said they're doing more of the physical part of it." (153)<br>Yes. She loves it." (153)<br><br>"When he was at his old school, it was just like you had your basic, old-fashioned jungle gym and that was it. At his school now, they had basketballs out there. Like a lot more equipment type stuff. Things to engage them." (095)<br>"Recess, yes, because they do walk. They require them to walk at least a lap before they go play." (096)<br>"And of course, there are things that with the playground, sometimes what I hear is that they can't play on the monkey bars…" (102)<br>"No." (143)<br>"I haven't noticed any. They were always, they always had recess and whatnot, anyway. So I personally haven't heard of anything." (156)
**D. Discussions of Food**<br>**D.1 Child Use of Go, Slow, Whoa (GSW) language**<br>**D.1.1 Parents observed children verbalizing and using GSW to make healthier food choices.**	"Well, one of my kids is, he's all boy and all kid, and he wants to live on candy, and macaroni and cheese, and chicken fingers. And then there's the other kid who, he was the one I was already quoting about constantly asking me, "Is it a whoa food? Is it a slow food?" (102)

	“Whoa foods are, they'll talk about desserts, and they'll point out all of the things that are in the pictures on their cafeteria wall. And they'll talk about cheese, or pizza. They're not all desserts. But they point out what's on the pictures. And I have to try to explain things like why the chocolate milk is fat free, versus the two percent milk, which has fat in it, and why the two percent milk is actually better, even though the chocolate milk is fat free.” (102)<br>“Those. Yes. She says that's a whoa food." (153) “That's a go food.” (153) “So she was constantly when we were out grocery shopping, she would go over these foods." (153)<br>“She goes through the different categories and points out, "Okay. That's go food. That's a whoa food." That sort of thing." (156)
**D.1.2 Some children do not use GSW or may have difficulty due to learning disabilities.**	“No, [he hasn’t mentioned GSW] but I know that he has a disability, so he may understand it cognitively, but to put it in a way back to you in a way you would understand it, he may not be able to do that.” (090)<br>“No, I haven't heard him mention that.” (095)<br>“No. He hasn't.” (143)
**D.2 Other Changes in Child Discussion of Food**<br>**D.2.1 Parents observed children generally requesting healthier options at home.**	“I know he really pays attention to what is a serving….or he’ll look at cookies. ‘Mom, if I’m going to eat this, I probably should eat this.’ So I mean, I’ve seen that kind of change” (090)<br>“She's always wanting more vegetables. We will go to the store; can we get this? Can we get that? You know, fruit, vegetable. Loves the fruit. Not so much the vegetables. Loves the berries.” (096)<br>“He hasn't said anything.” (143)<br>“I guess on occasion. I wouldn't say on a daily basis. But, yeah, on occasion there's comments on what we're eating and whatnot for different meals. And she'll make comments of how it affects her daily activity, or whatnot. " (158)
**D.2.2 Parents observed children requesting family members to make healthier food choices.**	“She even made a comment one day about the type of dressing we were using, because I'm a big ranch fan. And it's health-wise not the best choice. But it is my choice. And she made the comment, ‘Well, Dad, maybe you should try something else to see what you would like.’ So apparently at some point she's been educated on it's good to eat a salad, but depending on what you put on top of it can also ruin the healthy value of it." (158)

**D.3 Parental Understanding of GSW**<br>**D.3.1 Parental awareness and understanding of GSW varied.**	“Oh yes. I didn't even know what he was talking about. I thought he was making it up. I ain't going to lie.” (090)<br>“Yes. Mm-hmm. They have it well memorized better than I do." (102)<br>“Go is the good food, slow is the food you should have just every once in awhile, and the whoa food is like junk food. Stuff that's not good for you." (156)<br>“I've not heard that. No." (158)
**E. Changes in Teaching about Healthy Eating and Physical Activity**<br>**E.1.1 Parents generally observed teachers incorporating healthy eating assignments and physical activities within classes.**<br><br>**E.2 Energizers/Brain Breaks**<br>**E.2.1 Parents observed brain breaks increased physical activity and assisted kids to have fun, relax, and focus during school day.**	“Well, I know they use it a lot in their math classes and stuff. When they're converting, they use phrases that were about healthy lifestyles." (090)<br>“I think they talked about the go, slow, or slow, whoa. That kind of thing that we didn't do last year when I subbed." (096)<br>“And the activities, a lot of coloring pages of vegetables, which were clearly indicated to reinforce the nutrition aspects and activity aspects. Things about how to play, and things to actively, you know.” (102)<br>“No. I haven't heard anything about it.” (143)<br>“Little drawings they had done showing the different things in the program.” (153)<br>“It's something we were doing together.” (153)<br>“I think I've seen a little bit of stuff pulled into the classroom and in the library about different foods and the nutritional education part of that, I guess." (156)<br>“I've seen more papers, things that she's done in class she's brought home." (156)<br>“No assignments.” (156)<br>“No. I haven't. And honestly I'm not saying that's the school's fault on this program, or my child's fault, or anybody…. I don't get to have those daily conversations with her.” (158)<br><br>“Even when they go out on field trips and stuff, they still take their brain breaks.” (090).<br>“Mm-hm. I think – I don't know. All I know is about every 10 minutes, they get up and they move. They do jumping jacks.” (090)<br>“He does like it. He says that it breaks up the day because my kid is fidgety anyway and he said it breaks up the day and wakes him up and it's something different. My kid likes to dance around anyway." (095)<br>“We actually went to school during the night that they did this and we participated in that where we got up and danced with the kids and everything.” (095).

**E.2.2 Some parents observed physical activity used instead of candy/sugary snacks as a reward at school.**	"So especially the little kids, it gives them that little bit of wiggle time so they can settle down again. They really enjoy it." (096)<br>"They like to tell me about brain break and their Smart Boards. That's another thing. I had forgotten about that, the Smart Boards, all the dancing and songs and stuff they do on their Smart Boards and brain breaks. And Nicholas was acting one out just the other day. (102)<br>It was part of the how to wind down after doing an exciting and energizing activity. He was moving his arms like a clock to literally unwind." (102)<br>"No. I hadn't heard anything about it." (143)<br>They love them." (153)<br>"They have several different programs that they play along with GoNoodle." (153)<br>" The kids love those." (165)<br>"They get a break, and they get up and dance. They move, and sing, and just get a minute to recuperate, I guess." (156)<br>"But she's mentioned that all of a sudden they'll just stop doing what they do, and get up, and rotate their arms, or reach down and grab their legs, like some kind of fun game inside the classroom." (158)<br><br>"…instead of them giving a kid a reward of a lollipop, they bring in bouncy houses, things like that, where they're still active…" (090)
**E.3 Other Changes?**<br>**E.3.1 Parents positively responded to expanded healthy options in the cafeteria and healthy information provided by the school.**	"I know there has been some newer additions to the menu this year; the past year I think. So they have more choices and they will require them to have a fruit with lunch whether it be a fruit juice or whatever fruit they were offering. You know, some of the other choices were like yogurt and cheesesteaks and that kind of thing." (096)<br>"I know they walk during the day every day they walk laps around the track." "Even on PE days." (153)<br>"They have posters and things like that up throughout the school, and I've been there when they've done the announcements and they incorporate the little thing about nutrition or thing about moving or something in the announcements, I think, on Fridays." (156)<br>"Yeah. And the teachers dress down on Fridays." (156)<br>"My wife actually works there, too. She's a school teacher, as well. So I know, I hear a lot of things from her about how they're becoming a physically active school, in general." (158)<br>"Not that I can think of. " (096)

**F. Parental Views on CATCH changes and Observed Impact on Children/Family**<br>**F.1 Most Important Changes with CATCH**<br><br>**F.1.1 Parents expressed that increased movement/ exercise as well as focus on healthy eating are the most important changes from CATCH.**<br><br>**F.2 What is Not Working with CATCH**<br>**F.2.1 Parents generally viewed CATCH positively as working well, but suggest some change in the cafeteria food options.**	“I think moving every 10 minutes. That's just me.” (090)<br>“I can’t figure out if it’s the recess or the energizer." (095)<br>“I think it's the exercise." (096)<br>“I think the best answer I can give to that is, of course, the change that comes being a magnet school, and having that be the theme that is what makes it a magnet school, then it's such a well-chosen one, such a good one. But there's still such a small community, I think. It can only be a better experience for all the teachers, all the students, and everything to have it be a magnet school." (102)<br>“More activity.” (143)<br>“For the kids to try to find their healthy individually. So what works for them: food, exercise-wise. They found what works for them. Because if they can't find their individual medium, none of the information sticks.” (153)<br>“Just the extra focus on eating healthy and exercising and everything." (156)<br>“For me personally, I would say her eating habits right now.” (158)<br><br>“I don’t really see any.” (090)<br>“Maybe not having it in more schools." (095)<br>“I don't know that the menus in the cafeterias are the best." (096)<br>“I don't. I have no idea of anything that would not be working about the CATCH program." (102)<br>“I'm impressed with the whole program.” (153)<br>“I don't know. I don't know if CATCH is actually, if the – I don't know with the Obamacare and Michelle Obama's little thing in the lunch room, I don't know if it's done anything there. You know?" (156)<br>“ I don't know. That's a tough question because these kids are elementary…" (158)
**G.1 Changes in Child and Family Behavior**<br>**G.1.1 Parents observed that CATCH generally reinforced healthy eating at home, encouraged children to try**	“I wouldn't say it was necessarily because of that [CATCH], because we had started it before we enrolled him here, but it's made it easier for the children to accept when they're

**new foods and increased healthy food intake, such as fruits.**	hearing it from somewhere else, and it's not just mama's way..” (090)<br>“He is not as, ‘No, I'm not going to try that.’ He is more say, ‘Oh, okay. Yeah, I will try.’ Then he is like, ‘Oh, yeah, I really like that.’ He would not eat squash before. Now he is starting to eat squash." (095)<br>“I think Jacob has become a broader eater meaning he has—trying new foods, not as picky…..Drinking more water.” (095)<br>“He doesn't eat much of nothing any more.” (143)<br>“She drinks more water. I guess, yeah. More fruits and vegetables, but she's kind of always been that way, anyway." (156)<br>“The water instead of something else.” (156)<br>“I've noticed she, recently, I guess this past year, or maybe a little before, maybe started last summer, so to speak, she started eating a lot of fruits.” (158)
**G.1.2 While some families changed eating habits or stayed the same, others had challenges. .**	“I can't say that we have. We should, because we need to for ourselves anyway. We are always in a hurry. " (096)<br>“Well, I'm pretty big believer in that we have very healthy eating habits here. I always try to cook in the most healthy way I can with our own family situation, and as it is. And have the occasional treat, so it's not like they're going to go completely wild the minute they leave the house because they feel like they've been living on tofu.” (102)<br>“Number one, our serving sizes have gone down.” (153)<br>“It really brings your attention to it.” (153)<br>“And especially when you have a little eight-year-old on your right saying, ‘This is a whoa, Mommy.’" (153)
**G.1.3 Changes in physical activity after CATCH varied for families (Increased, Reinforced, Same, or no change).**	Maybe it’s a slightly enhanced it, but it’s something we kind of did anyway.” (095)<br>“I think, no. Although, there should be. “ (096)<br>“Yeah. I think that the fact that it's getting promoted so much. The awareness level has been raised.” (102)<br>“It’s about the same.” (143)<br>“We are getting at least 30 minutes of exercise a day.” (153)<br>“I don't know. We've always been pretty active.” (156)<br>“I mean, she's made that connection with gymnastics and being physically fit. So there's been some kind of connection made, and if it's coming from this program, that's great.” (158)

**H. Parental Recommendations on CATCH**<br>**H.1 Other Recommended Changes**<br>**H.1.1 Parents positively viewed CATCH and recommended increasing PA in CATCH and expanding CATCH to other schools.**	"I honestly would like them to do some more really local-based 5ks." (090).<br>"It's something that I think needs to be expanded and see if you can get it federally funded." (095)<br>"I don't think anything. I think everything else is pretty good." (096)<br>"I don't know. Seems great to me." (102)<br>"I don't really have anything right off the top of my head." (143)<br>"I would like to see more brain breaks during the day, maybe less time per brain break to spread them out a little bit." (153)
**H.2 Increase Family Involvement**<br>**H.2.1 More communication with parents and adding family specific activities for home and at school may increase family participation in CATCH**	"Yeah. More information sent home about it.[CATCH]" (156)<br>"Well, what the kids are doing, what the kids are learning, more in-depth information on the CATCH program." (156)<br>"So it's not only do they understand the program itself, I would maybe inform the parents a little bit about the program, what's going on at school so we can ask about these questions, or this program." (158)<br>"Well, the kids are supposed to be bringing stuff home, I don't guess there's any way to make sure they do. I don't know what the answer to that is exactly." (096)<br>"I haven't seen a lot of about the communities to be involved outside of personal family activity time, and cooking, and all that. But maybe it will change." (102)<br>"So we try to be involved. And I'd like to know how to be more involved." (102)<br>"Do more adventures. Like nowadays have the parents put, 'Okay. This is our adventure for the day, or for the week.'" (153).<br>"I don't know." (156)<br>"I guess, being involved in school." (156)<br>"Maybe have an activity day at the school, in general….Maybe have a CATCH day, just have something dedicated to that program that allows the parents to really understand it and maybe get involved a little more about it." (158)<br>"I think that people around here, it's just a lot of them are just low educational socioeconomics as well. Paying for

**H.2.2 Parents recognize that circumstances such as education/socioeconomic status may impact parental involvement**.	things for their kids to be in things, sometimes they can't afford it." (090)<br>"You don't know until you are told. A lot of illnesses in the United States or in the world is because of lack of knowledge. I think if people are informed, people are educated, and people know, then people will be better." (095)

Figure 3: Staff

Domains and Themes	Illustrative Quotes
**A. Background**<br>**A.1. Time at Stovall-Shaw**<br>A.1.1 Staff worked at Stovall from 1 to almost 16 years with most between 7 and 14 years.	"This is my 16th year." (87)<br>"Two years." (91)<br>"Fourteen years." (149)<br>"I've been there for 12 years now." (154)<br>"Nine years." (155)<br>Since "August 2016" (92) - less than a year?<br>"Just a year. This is my first year." (1)<br>"Possibly eight years; seven or eight years. Too long to count." (93)
**A.2 CATCH Role**<br>**A.2.1 While the majority of staff attended CATCH trainings, few staff members engaged in planning to bring CATCH to Stovall.**	"Actually, I was one of a few people that worked with the principal in researching it and kind of feeling out the staff here to see if they would be interested in implementing the program, and then we brought in the trainers. From there, me, as well as all the other staff members, participated in the formal training." (87)<br>"I didn't – I wasn't planning. I think I was hired the summer after. " (91)<br>"Well, I'm not a CATCH team member, but we had the training about the CATCH program and implementing the CATCH curriculum." (149)<br>"Well, I wasn't on the planning committee, but I went through the CATCH training and every classroom participates in the different parts of CATCH." (154)<br>"I attended workshops, and then I did lessons with my class." (154)<br>"None, no role." (92)<br>"I didn't plan anything. As a matter of fact, I didn't even know we was doing it. They just told us we was doing healthy and active living, but I didn't know it was particularly called a Catch survey." (97)

**A.2.2 Bringing to Stovall**	"I really don't. " (97)<br><br>"Well, I'm sorry. I did – I do help plan some of our menus. Does that count?" (93)"Well, we have government guidelines. We are kind of in line – I would say we are in line with the CATCH Program is that we do not have any salt anymore. We offer a fresh fruit every day, a canned fruit every day and two vegetable choices every day." (93)<br><br>"No. I didn't really. (149)<br>"No." (154)<br>"I was on the leadership team when we talked about bringing it, and so we just talked about things that we thought our community needed and wouldn't, I guess, be offended by and we could implement as a partnership. And so that's why we thought that the CATCH program would be one of the best programs out there because of our farming and just in our community, just without offending anybody." (155)<br>"I would say probably not." (93)
**B. Knowledge of CATCH**<br>**B.1 Majority of staff recognize the objectives of CATCH to promote physical activity and healthy eating**	"...we have a manual that we have lessons in, and ours is Hurray for Hill. Most of our lessons are centered around go foods and go activities." (87)<br>"I think that the program is mainly about educating children on the correct way to take care of themselves to live a healthy lifestyle, which would include making good food choices, and choosing to exercise. That's probably my best summary of it." (87)<br>"To promote healthier living, the healthier food choices." (91)<br>"I know it's a program that's designed to help children learn about healthy living, food choices, exercise, and making better choices so that when they grow up they'll continue to make those healthy choices, hopefully." (149)<br>"Teaching kids how to have a healthy lifestyle." (154)<br>"That we're increasing our movement, and that it has positive effects on our brains, and our overall awareness of how movement makes us happier and holistically better people." (155)<br>"Honestly, I don't really know too much. I know it's the health and wellness, but that's about it." (92)<br>"Nothing much, just healthy and active living. That's all I really know about it" (97)<br>"I did take the seminar class along with the staff and I saw where the exercise program was implemented into it as far as the nutrition part of it." (93)

**B.2 CATCH at heart of Magnet school**	"I know that we became a magnet school and our draw, or what we were trying to get people to come to our school for is we were trying to get kids to be healthier, to eat healthier, to exercise and just make it part of their everyday lives. And we were using the CATCH program to accomplish that." (154)
**C. Observed Changes at School**<br>**C.1 PE**<br>**C.1.1 Staff observed increased physical activity, variety of sports/exercise, and incorporation of GSW in PE since CATCH**<br><br>**C.1.2 Recess**	"I think that they are more active and they're constantly participating. There's not a lot of sit time that they're waiting for other people to take turns anymore, that the activities that the PE teacher has incorporated has everyone moving all of the time, and there seems to be a variety. " (87)<br>"Not really. I hear them talk about different things that they do. I don't know that I knew before. I don't know what the PE classes were before." (91)<br>Well, PE's been pretty active, but I do see that there is more action now than it used to be, I do feel like." (149)<br>"I really don't observe the PE class. And of course, I don't teach that." (154)<br>"Well, I think they have different, like they have a board that says like the go, whoa, and slow foods." (155)<br>"They have that up there. And I've seen prints. He's put different facts up that they can see when they come in. I don't know if they discuss it, but I see it." (155)<br>"Yes, my class – students are taking PE once or twice a week. So yeah, they are very active." (92)<br>"They do relays. They do obstacles. They do walking, running, exercises like jumping jacks, windmills and things of that nature." (92)<br>"Okay. So in PE, I really like it that Coach Holmes, he never… Basically he does – there is not really a winner. You know when you play racing games, the first one, the first team that wins, they sit out. He never had our kids sit out. " (97)<br>"That's different [from PE in prior school]. That's where a lot of my questions came from. Does the winner have to sit down? He would say no…." (97)<br>"That I wouldn't know about." (93)<br><br>"We have 30 minutes of recess every day." (154)<br>"And every day when they go out for recess, the first thing they do, they know. They walk two laps, and it's part of our fitness program." (154)

**C.2 Changes in Food Conversation**<br>**C.2.1 Staff observed that students are identifying GSW foods and requesting healthier food options at home.**	“That's mainly the way that they refer to them now. Those are – that jargon has become such a part of their daily conversation….Yeah, when they're talking about it, or if we ever have a snack in class, they'll say, "Oh, this is a whoa food." (87)<br>"I'd hear them at lunch, "Oh, this is a go food. This is whoa food." So I'd hear them talking about that. They'd see me, "Oh, you're eating a go food." Or "That's not". So they do refer to that and talk about that." (91)<br>“...we talk about like the go food, and the slow food, and the whoa foods. And they try to make – they're conscious at lunch. They're like, "Look… I got some go food today." And, "Look …I didn't eat all my french fries today because I didn't want – ‘ you know.” (149)<br>“He won't – my husband loves fried chicken. I'm just going to be honest. And my son has gotten where, ‘Daddy, I don't want fried chicken. I want my chicken grilled. Please grill mine.’ You know?" (149)<br>I've heard them discussing going through what's on their plate, especially towards more at the beginning of the year, because everything's just kicking off and getting fresh in their minds again." (154)<br>“And I've heard other kids, like if they make a choice to get a cookie, and then they sit down and they're eating their cookie before they eat their lunch, I've heard other kids say something to the other saying, ‘You shouldn't be eating that before you eat your other food.’" (154)<br>“I actually hear my students, the ones that bring school lunch, talking about what they have in their lunchbox, and how they help their parents plan for that." (155)<br>“So like I said, I've actually had a parent speak up at one time because her son came home and said that he was kind of confused and he just said, "I'm not allowed to eat that at school." And so he took it the wrong way, you know, as it was, and said, "I can't have it." Well, then the mom, of course, got mad." (155)
**C.2.2 Teachers observed some misunderstanding about GSW concepts at younger ages.**	“If broccoli is green, you can eat it. Yellow foods like a banana, you shouldn’t eat a lot of. They were like, the red foods you shouldn’t eat it at all. They were looking at it more what color the food is to go along with the color.” (92).<br>“Yeah, that was my pre-K kids." (92)<br>“So me, I was going to say my kids don't really talk. We<br>work a lot with the regular pre-K and they will talk about it.<br>They will talk about what are good grains." (97)

	GSW: “Yes. I am familiar with that. We have those posters hanging up and she also, on her weekly things, she will implement that as well. We have them hanging up near the cafeteria." (93)
**C.2.3 Teachers positively want to model healthy food eating.**	“I think this has allowed us to bring it down to even the youngest children, and really get them started earlier. We watched more short video clips this year about different aspects of health, whether it being making food choices, or dental health, things like that. We've been able to tie those subjects into the CATCH program and relate it back to, again, taking care of yourself, making the good food choices, and being active." (87)<br>“It brings that awareness where sometimes it’s hard for, it makes you aware as a teacher too, and as a person. Even in my own life., ‘Oh, what do I need to do?’ because I’m modeling this behavior. Am I making this choice if I’m telling them that?’” (91)
**C.3 Changes in Healthy Lifestyle Teaching**<br>**C.3.1 Teachers appreciate CATCH lessons are short and incorporate them into classes (science)**	“I like how short they [CATCH lessons] are.” (155)<br>“It's in addition, and they [CATCH lessons] don't take long. So it's easy to put in between like if you have something special going on one day and you know you can't get your math lesson in, so then that's something small that you can fit in,...Your time's not wasted." (155)<br>I try to integrate it [CATCH lessons] some in our units that we have. Sometimes I read a lot because it's easier to do, but yeah, we try in science. We try to integrate that as much as we can.” (91)<br>Now, we run the CATCH lessons through the science class." (154)<br>“With fourth and fifth grade. And I don't teach that. Chris Leas teaches that. So in my classroom, I just reinforce the healthy, just the talk and stuff that goes along with CATCH,...” (154)
**C.3.2 Teachers teach nutrition, such as food labels, and incorporate PA into classroom lessons.**	“And reading food labels. I had a lot of children – we just, a couple of the lessons about food labels and stuff. And they really pay attention to that, and they show it to their parents, and they talk about it with them.” (149)<br>“No, I haven't had the opportunity to do that or to see that." (92)

	“So…when I was teaching shapes and numbers, I did a game called Musical Shapes….I would try to make lessons geared towards getting up and moving and not just sit and get kind of thing.” (91)<br>“I incorporated healthy eating into my lessons because for pre-K, we have to follow a certain guideline for what they eat anyways. We will discuss which foods are healthy, which foods are less healthy, but I have seen children around the halls reading the slow, go, and whoa foods. The red, green, and yellow foods. I have seen kids looking at those posters and reading that." (92)<br>“No, I haven't. Me and the PK teacher, we kind of stick to one another. I don't really… No, I haven't really heard too much about that.” (97)<br>“No. I did give out some information pamphlets that I had received from the government on the farm to school produce and fresh fruits and stuff that we get from them." (93)<br>“Because they said they were doing nutrition lessons in the classroom. I do know that they have done – I know my lower grades have done one on the strawberries and where they come from and all that." (93)
**C.4 Energizers/Brain Breaks C.4.1 Staff offer a variety of activities during brain breaks that students enjoy and observe that Brain Breaks keep students active, engaged, and improves their focus.**	“Well, the way that I incorporate energizers, and we call them brain breaks too, into our classroom, it's a variety. Some are from an internet site that we found called GoNoodle, and they really love those because it incorporates silly songs, and some of them are dance routines to popular songs that they know. It's kind of relatable to them. " (87)<br>“So Fridays, being able to wear tennis shoes and say yoga pants, or a t-shirt, it allows us to be able to do these things more freely, with our students.” (87)<br>“MyKids love them.” “[W]e do a lot of GoNoodle.” (91)<br>“We do the brain breaks. We try to do them whenever we see them moving around, whenever we see them kind of losing interest is more or less what I do.” (149)<br>“And they look forward to them, too.” (149)<br>“but also we do every 30 to 40 minutes the energizers, the brain breaks, where they get up and exercise.” (154)<br>“That became part of our thing that we do at Stovall that – I mean, that's just part of our magnet school now. That's one of the things that we talk about that we do, I think, that works very well. It definitely keeps kids from getting sleepy, or tired, or bored. And I like it myself." (154)<br>“We do exercise videos for the most part. For my pre-K kids, that's what we do – exercise videos.” (92)

	“It does help them focus very much.” (92)<br>“…everybody is required to do brain breaks and things like that. We have to explain to our kids why we do the brain breaks, why we stay active.” (97)<br>“If you give them a break from something and give them time to – like for our kids, for specialty kids, we have to offer them breaks because sometimes it becomes too much. So I think if you allow kids to do that, they can learn better. They can get the wiggles out and stuff like that.” (97)<br>“No, I do know the kids like the ones because I actually used it in an afterschool program last year because my teacher had access to the… Is it the smart board?" (93)<br>“We used that a few times and the kids actually watched the little videos that were on it." (93)
**C.4.2 Teachers positively associate Brain Breaks with increased activity for students at home and staff at school.**	“So we, that's something we do all the time. We use GoNoodle. We do different games that we learned with the CATCH lady that came and taught us little games to play. So there's interactive little moving activities. And they're quick. And it's kind of neat because the kids, now, they do them at home. So during parent-teacher conferences, I have a lot of parents say that their child gets on GoNoodle and they do the specific activities." (155)<br>“As a teacher, I used to think that teachers were very active. Well, if you really really really really think about it, teachers sit and read a book….But this gives me an opportunity to get up and wiggle, too." (155)
**C.5 Fit Friday**<br>**C.5.1 Staff express that teachers and students wear fitness clothes and engage in more physical and CATCH focused activities on Fridays.**	“For Friday, I try to do – we try to do more laps on Friday around the track, or I do more songs in my classroom depending on the weather too." (91)<br>“When she does the announcements, she'll give us a Friday tip. And she usually about different things, about getting out and exercising, or about foods. The children, they listen. They really do listen to what is being said. And they pay attention…." (149)<br>“That's like a day that we really set aside to just really try to reinforce CATCH things. Things that we learned in our training and stuff, and just making sure that it's a day that's set aside to focus on fitness and really talk about it with the kids more on that particular day. And the teachers love it because they get to *[Laughter]* wear fitness clothing." (154)<br>“So fit Friday, everybody wears something that gives them a little bit more ability to move, like as far as teachers….And then the GoNoodle that we choose is different." (155)

**C.5.2 Fit Friday has positively influenced other teacher events such as "Fat Friday".**	"We're able to wear fitness clothes, yoga-style clothes. Kids can wear those kind of clothes as well. We incorporate extra physical activity throughout the day." (92).<br>"Fridays, we get to come in… What is it? Yoga pants. We've got our "Working on Wellness" shirt. We are supposed to be talking about why we are doing this; to stay active and be well and stay healthy and why it's important." (97)<br><br>"We've even, on occasion, we have Fat Friday – I hate to use that terminology – once a month. Several months this year we have implemented healthy foods versus a lot of junk food." (93)
**D. Additional Staff Observations**<br>**D.1 Other Changes**<br>**D.1.1 The cafeteria has added healthier food choices and implemented methods to limit food waste.**<br><br>**D.1.2 Teachers have moved towards activity- based rewards for students.**	"Okay, well I was going to say that our cafeteria has changed some of their food choices. They have more choices now than they used to in the cafeteria." (87)<br>"I know in the cafeteria they put bulletin boards up that are highly engaging and they go along with our curriculum. I know at the lunch tables they have baskets, and so if a child does not want to eat their fruit, like an apple or a banana, not like a cut-up apple, or apple boat, but they can put it in the basket and a child can go over to the sink and wash it, and they can eat that if they want it, like if they want more food." (155).<br>"I mean I think there's some more with the cafeteria. Changes – if you're not going to eat that food, put it in the basket in the middle or put it up there on the counter, so we don't throw it away." (91)<br><br>"We also have behavior rewards monthly for kids that have – maintain good behavior. This year, that committee has really worked hard to make the incentives and the rewards activity-based. So rather than have an ice cream party, they've had a dance or extra recess." (87)

**D.1.3 Staff has implemented Wellness Wednesday and Family Fit Day during the school year.**	“Well we did form a CATCH committee at the beginning of this year,....This year that committee got together and really made an effort to make Family Fun Day more of a Family Fit Day." (87)<br>“Or the wellness Wednesday? They're, yep.” (149)<br>“Well, it's kind of like where she does an extra health tip or gives us some information about maybe a famous person that has made good choices and healthy choices, or just kind of something like that.” (149)<br>“We did a – a fit – a community… What is it? A fit – they have to correct me every time. I think it's called a Family Fit Day. " (97)<br>“I like that. … We had a Zumba section. We had yoga. We had something going on in the gym and it was where families came out. They ate and stuff. What they did was fun because it was like a carnival, but it was really fitness based. The food that we served wasn't bad food." (97)
**D.1.4 Staff and Parent Teacher Organization (PTO) are providing healthier food options for staff.**	“No, I do know that when we had our teacher appreciation week, our PTO served a lot of healthy breakfast items; the granola bars, the breakfast blueberry type bars, fresh fruits….it’s a lot more healthy things to choose from than the heavy doughnuts and stuff like that.” (93)
**D.2 Most Important Change**<br>**D.2.1 Staff members expressed that physical activity from Brain Breaks and Fit Friday, Go, Slow, Whoa lessons, and the changes to expand choice in the cafeteria, are the most important changes.**	“I think well, I guess my thought is a combination. It would be specific lessons on the go, slow, and whoa foods, and the changes in the cafeteria…" (87)<br>“Probably teaching at such a young age. Because I don't know – sometimes at school, it's not in the curriculum. I mean we have a health curriculum, but it's just not pushed as much." (91)<br>“I don't know. That's hard to say. I feel like the implementing the brain breaks and the more active learning component have been important. I also feel like the nutrition component and the cafeteria, where we have more salads and things like that available, or fresh vegetables and fruit choices than we used to have. I think that's been important, too, because the children are making better choices." (149)<br>“The brain breaks in the classroom." (154)<br>“Because I've definitely seen a positive impact on the kids as far as their, like I said, time management, they're not bored, they're not falling asleep in class, they're not zoning out. I just, I've really enjoyed that as a teacher, and just seeing that impact has had on them." (154)<br>“Just the simple awareness, just the awareness of what we're doing with our bodies and what we're putting in our bodies." (155)

	“Just actually taking in more physical activity, the whole Fit Friday thing. I think that's very important.” (92)<br>“I feel like it gives the kids something to look forward to, like if you know you're going to do extra fitness stuff on Friday, you may do certain stuff other days of the week and not even realize it just because you're used to doing it on a Friday. It becomes incorporated into your routine permanently. " (92)<br>“That is a tough one. I see, even within myself, because we pre-K teachers, we talk about it all the time; working there has made me more conscious of what I eat.” (97)<br>“And the – like I said, the Wellness Wednesdays, especially Friday… I got excited to wear yoga pants. … So I would say, even for me personally, I know that it made me more conscious of what I'm eating; how am I in taking food.” (97)<br>“I don't know if I can honestly answer that because on my end, I only see my end." (93)
**D.3 What’s Not Working**<br>**D.3.1 Most staff members believe that CATCH implementation is going well at Stovall.**	“Hmm. I don't see anything that's not working. I feel like the whole school and a lot of the families are making better healthy choices and encouraging each other to be healthier. I don't really know of any. I think the hardest thing is the carry over into the home." (149)<br>“I can't tell you anything that's not working, because I think everything's working with it, about it." (154)<br>“Well, again, on what I see on my end and what she's announcing on a weekly basis about the Catch Program and then all the stuff that we've implemented, from what I can see on my part, I would say that I think it's done well." (93)
**D.3.2 Some staff believe more can be done to involve the home and community, more training, and provide more food choices that are consistent with CATCH lessons.**	“I think we can do a better job of involving Parents and the community.” (87)<br>“Well, I think for me it's hard because I didn't have that prior training. I don't know if it's not working, but it's hard for me." (91)<br>“I think it's Catch could touch a little bit of lunch – I'm not saying take away children's – we eat cookies…I think if we are supposed to promote health and wellness, our food should be better." (97)<br>“What would I say is not working? I don't think anything is necessarily not working, but if I was to give a suggestion, and it kind of goes a little bit with me being a parent, is that I know my – and I don't know if this is part of the CATCH program, necessarily – but I have a second-grader, and she would come home about once a week talking about some different foods that she tried, and they were all like

	vegetables, and fruits, and it was just simple things like, "Mom. My teacher told me I could put my fruit in my yogurt." So she came home and she was like, "Can I do it?" And so that's what she ate for breakfast for the next two weeks." (155)<br>“I don't know. The simple fact that I'm in pre-K, and we have to follow certain dietary guidelines, so not getting around to everybody as well as to some other age groups." (92)
**E. Staff Recommendations**<br>**E.1 Desired Changes**<br>**E.1.1 Staff recommend to improve communication and information to Parents and Community about CATCH.**	“Parents, letting parents know and getting their involvement because I think it would be – I mean obviously the kids are going to go home and share something. Some parents asked me, "What does this mean?" Their kids might have talked about it, but they weren't sure about it.” (91)<br>“I would like to see it continue, continue to have the lessons, and continue to have the access to the materials, and the play equipment, and the – we have this sign when you go into school. " (149)<br>“And we've discussed it, and talked about it, and trying to figure out ways that we can get it out in the community more. Unfortunately, it's just when we have parent participation night, it just seems to be, it's like the same parents always come for everything. And it's, unfortunately, the parents that probably really don't need the training.(154)
**E.1.2 Staff recommend expanding CATCH and the CATCH committee.**	“I think I would like to see that committee continue to grow that focuses on the CATCH curriculum and active activities that relate to the CATCH program." (87)
**E.1.3 Staff recommend expanding healthy food options, healthy food tastings, and decreasing less healthy options at school.**	“Well, I'm just going to throw this out there. It would be awesome if we could have a salad bar at our school. " (155)<br>“Yes, cookies are offered every day in the cafeteria. If you have money, you can buy them, or chips. You can buy that stuff in addition to what you already have for lunch. I think it can get excessive to be offered every day." (92)<br>“I would like to see, maybe like instead of Catch, just telling us… What is it? The slow. The no, slow, or whoa food. I'm sorry. The go, slow, whoa. Why not provide it once a month? The food that we are supposed to – for the kids or even for the teachers." (97)<br>“On my behalf, less lunchboxes." (93)<br>“[F]rom what I can tell, they’ve implemented the things that I saw in the meeting with a seminar that we went to.” (93)

**E.2 Ways to Increase Staff Engagement**<br>**E.2.1 Staff recommend creating physical activities and CATCH promoting events to build comradery among staff.**	“I think in addition to becoming part of the committee, I would say that maybe the staff – it would be good if we had groups like say maybe an aerobics class one week after school, or a walking club that walks after school, or before school one morning. Just a way for the staff to build that comradery and commitment to the program, and then it would – the students would notice that even more.” (87)<br>“So for Fat Friday, when we want the bad food; so making a committee for that. How about instead of a Fat Friday… You know, because that's what we call it at the end of the month. We have different – what do you call it? Potluck. People bring different things." (97)<br>“Why not have a healthy Friday or that something Friday." (97)
**E.2.2 Staff recommend increasing time and support for planning, and encouraging staff collaboration on, CATCH to improve staff engagement.**	“I think sometimes giving us time, even to sit down. How do you implement this in your class?" (91)<br>“Or maybe talking with other teachers. How does it work for you? What can I do? Or what did you do? And share with each other." (91)<br>“And I think just keep continuing to have a CATCH team that is just giving us little checklist and stuff to make sure that we're covering everything and continuing to do what we should do. I think that's a good way." (149)<br>“Me, I'm really not involved at all. I don't know. Maybe going into other classrooms to see what they're doing, so that I can be part of the same thing, collaborating more with each other." (92)<br>“I guess I could write a grant. I know there's several different grant opportunities out there. I just haven't had much luck with getting any grants recently. But write a grant, or even ask the parents to bring stuff in." (155)
